# Qigong mind-body program for caregivers of cancer patients: design of a pilot three-arm randomized clinical trial

**DOI:** 10.1186/s40814-021-00793-4

**Published:** 2021-03-19

**Authors:** Pinky Shani, Kristin Raeesi, Eli Walter, Kai Lewis, Wanyi Wang, Lorenzo Cohen, Gloria Y. Yeh, Cecile A. Lengacher, Peter M. Wayne

**Affiliations:** 1 University of Houston, College of Nursing, Houston, TX USA; 2 Texas Woman’s University, College of Nursing, Houston, TX USA; 3 Houston Martial Arts Academy, Houston, TX USA; 4 The University of Texas MD Anderson Cancer Center, Houston, TX USA; 5 Beth Israel Deaconess Medical Center, Harvard Medical School, Boston, MA USA; 6 Osher Center for Integrative Medicine, Harvard Medical School and Brigham and Women’s Hospital, Boston, MA USA; 7 University of South Florida, College of Nursing, Tampa, FL USA

**Keywords:** Cancer caregivers, Qigong, feasibility, Randomized control trial, Study protocol, Internet, Distress, Quality of life

## Abstract

**Background:**

Informal caregivers, often family and friends, experience significant psychological and physical distress leading to reductions in health and quality of life (QOL). Mind-body interventions focused on caregivers are often limited and do not address multiple barriers, including caregivers’ economic, geographic, and time constraints. Translation of in-person, community-based interventions to Internet-based delivery may offer greater accessibility for caregivers, leading to increased adherence.

**Methods:**

*Caring for Caregivers with Mind-Body * implements a three-arm, pilot, randomized controlled trial to evaluate the feasibility of delivering a Qigong intervention (Eight Brocades) to cancer caregivers. A total of 54 cancer caregivers will be randomized into one of three 12-week programs: (1) community-based Qigong, (2) Internet-based Qigong, or (3) a self-care control group. Study-specific aims include (1) modify intervention content for online delivery, (2) evaluate the feasibility of recruiting and retaining cancer caregivers into a 12-week clinical trial, and (3) evaluate the feasibility of collecting and managing data, and the suitability of questionnaires for this population. Several outcomes will be assessed, including caregiver QOL, caregiver burden, caregiver distress, perceived social support, physical function, and cognitive function. A 6-month follow-up will also assess longer-term changes in QOL and psychosocial well-being.

**Discussion:**

Findings will be used to inform the design and conduct of a large-scale comparative effectiveness trial evaluating caregivers who received Qigong training delivered through community-based vs Internet-based programs. A finding that either or both programs are effective would inform care and options for caregivers.

**Trial registration:**

NCT04019301; registered on July 15, 2019; clinicaltrials.gov

**Supplementary Information:**

The online version contains supplementary material available at 10.1186/s40814-021-00793-4.

## Introduction

### Cancer caregivers are a large and growing population

Improvements in the detection and treatment of cancer have resulted in an increasing number of cancer survivors, with estimates of over 16.9 million in January of 2019 in the USA and projections of up to 22.1 million by 2030 [1, 2]. It is estimated that more than 1.8 million new cancer cases will be diagnosed in 2020. In the USA, there are approximately 43.5 million informal caregivers, with 1 in 4 caregivers spending 41 h or more per week providing care to an adult or child [3]. Informal caregivers are often family members or friends, responsible for caring for individuals with a variety of burdensome conditions including advanced age, dementia, and cancer. These caregivers provide 70 to 80% of care for those with cancer and are involved during the cancer care trajectory from diagnosis to death [4, 5]. The burden of caring for someone with cancer can be extremely high; 50% of cancer caregivers report increased levels of stress and depression with 40% indicating that they need help managing their own emotional and physical stress [6]. Additionally, research on the caregivers of psychologically or physically ill patients showed decreased caregivers’ quality of life (QOL) directly impacted the QOL of the recipients of their care [7–9]. Moreover, the burden among cancer caregivers often persists for years after patients’ initial cancer diagnosis, with evidence of long-term detriments to health and QOL [10–13]. Therefore, it is vital to develop effective and practical interventions to prevent and manage the psychological and physical stressors that reduce QOL in caregivers.

### The burden experienced by caregivers is complex and best viewed through a biopsychosocial framework

Caregivers burden is defined as “the extent to which caregivers perceive that caregiving has had an adverse effect on their emotional, social, financial, physical, and spiritual functioning.” [14] This definition emphasizes the multidimensional and biopsychosocial complexity of caregiving [5, 15]. Examples of psychosocial symptoms experienced by all caregivers, including cancer caregivers, include increased anxiety, depression, isolation, lack of social support, helplessness, loss of control, and fear of recurrence [11–13, 16–18]. More behavioral and somatic concerns include lack of exercise, poor sleep, fatigue, weight gain or loss leading to impaired immune system function, coronary heart disease, and early mortality [19–22]. Caregivers of both older children and adult patients also report a significantly higher prevalence of musculoskeletal pain, attributed to lifting and transferring heavy loads [23, 24]. From a biopsychosocial perspective, these symptoms are highly interdependent. For example, poor sleep and chronic fatigue are known to contribute to risk of depression, and depression and chronic pain are both linked to common inflammatory pathways [25]. This interdependent constellation of symptoms underlying caregivers’ distress has led to exploration of integrative, multimodal mind-body interventions that can address a range of psychosocial and physical concerns [26–29].

## Background and rationale

### Mind-body therapies for caregivers––gaps in the current evidence

Mind-body practices that target both psychological and physical dimensions of distress offer a promising and pragmatic therapeutic strategy for addressing the needs of caregivers [27, 30, 31]. However, the evidence required to guide such an approach is still limited in multiple ways. First, while a growing body of research supports mind-body practices such as Tai Chi, Qigong, yoga, and meditation for a range of symptoms in patients with chronic disease, including cancer [32–37], few large-scale studies have evaluated the effects of these practices in caregivers. Of the studies that have evaluated caregivers (mostly yoga or MBSR), many have utilized interventions tailored to patient-caregivers dyads [35, 38–41]. Although this approach has merit, it may limit specifically addressing caregivers’ psychological and physical needs. Additionally, while many MBSR and yoga studies show positive effects on mental health, most do not include physical function and disability measures. A recent study using a Qigong intervention for both caregivers and cancer patients found caregiver fatigue and well-being levels to be improved after a single Qigong class [42]. Lastly, more widespread access to mind-body interventions targeting caregivers has been challenged by economic, geographic, and time barriers [43]. Common barriers to in-person group classes (e.g., issues with caregivers’ travel to community-based programs) might be overcome with Internet-based delivery of interventions, offering a more convenient way for some caregivers to access the programs and increase adherence. While Internet delivery of individual-based mind-body practices is increasingly studied and shows promise [26], this approach has not been widely explored in caregiver populations.

### Qigong as a promising multimodal intervention for caregivers

Qigong is an increasingly popular multimodal mind-body practice that shows promise in addressing a broad range of psychosocial and physical factors highly relevant to caregivers. Sharing many characteristics with Tai Chi, Qigong incorporates elements of slow gentle movement, breath training, and a number of cognitive skills, including heightened body awareness, focused mental attention, and imagery—which collectively may afford greater benefits to health compared to unimodal therapies [44, 45]. In contrast with typical Tai Chi choreography, some Qigong regimens focus on simpler repetitive movement phrases, making them easier to learn through in-person instruction and especially via video-guided instruction. A robust evidence base across multiple adult populations suggests that Qigong and Tai Chi training delivered in groups can improve multiple domains of physical and emotional health, including those highly relevant to caregivers such as depression [46–48], anxiety [46, 48], poor sleep [48–51], musculoskeletal strength [49, 51], balance during functional activities [49, 51], pain [46, 52–55], and core underlying physiological processes such as inflammation [56, 57]. Reduction of overall distress and improved long-term prognosis is also supported by improvements in broader constructs including overall QOL [46, 48] and self-efficacy [27, 49, 58]. While Qigong is increasingly being used to help manage health and distress in caregivers, including at leading academic medical centers like MD Anderson Cancer Center and Harvard’s Dana-Farber Cancer Institute, very few studies to date have evaluated Qigong for cancer caregivers.

### Limited access to widespread use of Qigong for caregivers

A critical challenge in implementing any intervention is the practical issue of adherence and access [26, 59, 60]. Prior studies among informal caregivers, including exercise, psychotherapy, and medication, show low adherence [26, 60, 61]. A review of literature of web-based interventions for cancer caregivers found that caregivers are often reluctant to participate in support services due to long travel times or the feelings of stigmatization associated with participation in face-to-face support groups [62, 63]. In the case of Qigong, one possible solution is the Internet or virtual delivery of instruction. This approach would address the broad issue of access to evidence-based programs and would also provide an option for caregivers who cannot leave the home and/or allocate time required to travel to and from regular classes, often as a consequence of their caregiving duties. Of note, an analysis of the 2012 National Health Interview Survey data indicates that a significant proportion of the US population that report using Qigong and Tai Chi for health preferred self-directed learning from DVDs and Internet resources [64].

While a handful of studies support the potential for web-based or DVD-based learning of mind-body practices, evaluations of such programs have not been well tested, especially in caregivers. In a small feasibility study, Wu and Keyes delivered a 15-week-long Tai Chi program for older balance-impaired individuals using an Internet-based live-video conferencing platform [65]. They reported good adherence (average 78%), comfort with navigating technology, and high interest in ongoing training. A follow-up study compared the effectiveness of Tai Chi delivered via live-video conferencing, in-person community-based classes, and home-based self-directed video learning [66]. While all three groups showed trends towards improvements in QOL and multiple measures of balance and function, protocol adherence and improvements were lowest in the self-directed video learning group. Collectively, these studies support the promise of mind-body programs being offered remotely, but perhaps suggest that at least some live contact and support from instructors may be critical for obtaining higher levels of adherence.While a handful of studies support the potential for web-based or DVD-based learning of mind-body practices, evaluations of such programs have not been well tested, especially in caregivers. In a small feasibility study, Wu and Keyes delivered a 15-week-long Tai Chi program for older balance-impaired individuals using an Internet-based live-video conferencing platform [65]. They reported good adherence (average 78%), comfort with navigating technology, and high interest in ongoing training. A follow-up study compared the effectiveness of Tai Chi delivered via live-video conferencing, in-person community-based classes, and home-based self-directed video learning [66]. While all three groups showed trends towards improvements in QOL and multiple measures of balance and function, protocol adherence and improvements were lowest in the self-directed video learning group. Collectively, these studies support the promise of mind-body programs being offered remotely, but perhaps suggest that at least some live contact and support from instructors may be critical for obtaining higher levels of adherence.

## Materials and methods

### Objectives

The overarching objectives of this pilot study are to determine the feasibility of conducting a randomized clinical trial (RCT) evaluating community-based and Internet-based Qigong programs for cancer caregivers and to collect preliminary data on these programs’ impacts on QOL and a battery of patient-centered outcomes related to psychological and physical function. Findings will be used to inform the design and conduct of a fully powered trial. Accordingly, the primary aims of the study are to:

i. *Modify intervention content for online delivery*: A detailed 12-week Qigong curriculum based on the Eight Brocades, tailored to adult cancer caregivers, will be systematically developed for online delivery in collaboration with expert Qigong instructors.
ii. *Evaluate the feasibility of recruiting and retaining cancer caregivers into a 12-week clinical trial*: We will assess *recruitment feasibility* by the number of screened and eligible participants and the number refusing to participate. This domain of feasibility will require the following: (a) ≥ 50% screened individuals are study eligible and (b) ≥ 5% of eligible participants are willing to consent. With a goal of *n* = 54 enrolled over a 1-year period, we will need to screen 14 participants per month. *Participant retention* will be deemed feasible if loss to follow-up is ≤ 20%.
iii. *Evaluate the feasibility of collecting and managing data, and the suitability of questionnaires for this population. Several outcomes will be assessed including QOL, caregiver burden, caregiver distress, perceived social support, function cognitive function, and additional measures as listed in* Table 2: All outcomes will be assessed in-person at baseline and at 12 weeks, following completion of the 12-week intervention. A longer-term 6-month follow-up will assess a subset of outcomes using a questionnaire packet delivered electronically.

### Design overview and study setting

**Fig. 1:**
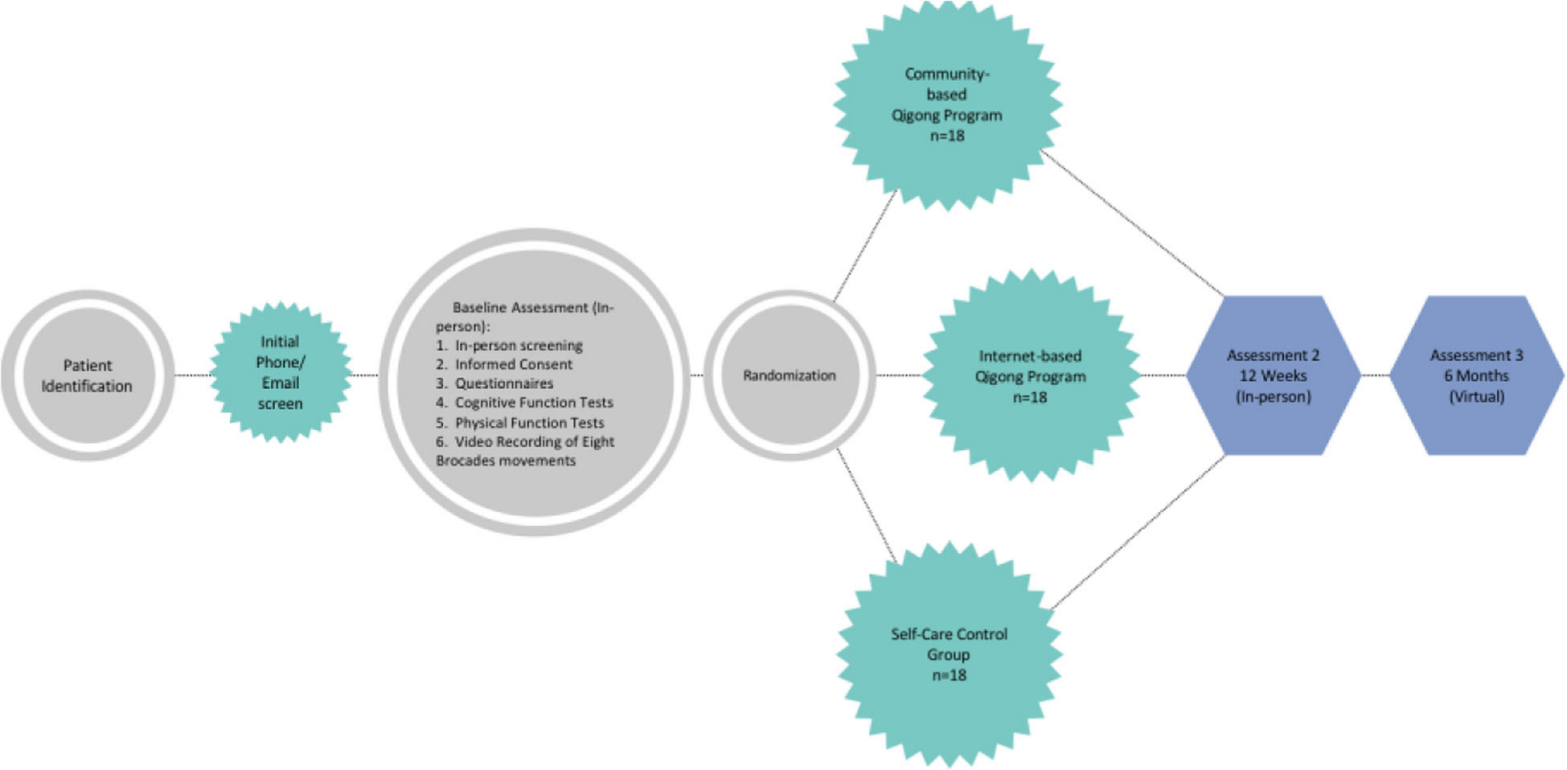
Study design and participant flow

The design implements a mixed-methods, pilot RCT with parallel allocation to the community-based group, Internet-based group, or self-care control groups. Participants who are interested in participating in the study will complete an initial phone screening to determine their eligibility. Eligible cancer caregivers will be invited to University of Houston campus for their baseline visit. At this visit, participants undergo formal consenting following University of Houston and National Institute of Health guidelines. Consented participants then complete the initial self-report assessments, cognitive and physical function tests, and will be randomized to one of three groups. Participants continue to receive the assigned intervention for 12 weeks. Follow-up assessments will be completed at 12 weeks and 6 months post-baseline. Participants receive a total of $125.00 in gift cards for completing the study. An overview of participants’ flow is provided in Fig. 1.

### Study population and eligibility criteria

This study is recruiting participants who are over 35 years old and currently a spouse, partner, family member, or friend providing physical, emotional, and/or financial support for a cancer patient. Participants must be willing to be randomized to any of the three study groups, complete study procedures, and read, understand, and speak English. All participants must have scored at least 3 (0 to 10 scale) on the National Comprehensive Cancer Network’s (NCCN) Distress Thermometer and be able to provide informed consent [84].

Participants will be excluded from the study if they have any unstable illness (e.g., recent hospitalization, unstable cardiovascular disease, active cancer) or psychiatric disorders (e.g., unmanaged depression or psychosis, substance abuse, severe personality disorder). Individuals with degenerative neuromuscular condition (e.g., Parkinson’s disease, multiple sclerosis); inability to walk continuously for 15 min; recent history of attending regular Qigong or similar (e.g., yoga or Tai Chi) classes defined as 20 or more classes in the past 6 months; or participation in more than 240 min of moderate-intensity exercise per week (as these individuals already exhibit high exercise self-efficacy and are less likely to be otherwise symptomatic) will be excluded [85, 86].

### Ethical oversight, participant recruitment, randomization, and blinding

This study has been approved by the University of Houston Institutional Review Board (IRB) with approval at MD Anderson for participant recruitment. Eligible participants will be approached at MD Anderson Cancer Center (MDACC) or local community cancer caregivers support groups for potential study inclusion. Our primary recruitment site is MDACC. Study staff meet regularly with physicians to inform them about the study, and brochures and flyers describing the study will be distributed to cancer caregivers via advertisements within MDACC and with cancer caregivers support groups. They also present the study to patient support groups within MDACC and post information about the study on relevant hospital websites, including those specific to recruiting volunteers into clinical trials. In addition, potential participants who meet the inclusion criteria will be recruited through non-hospital online websites and social media, including the Houston Chapter of the Oncology Nursing Society and the Family Caregiver Support Network. Other strategies include targeted advertising and flyers in public places (e.g., library, local stores). The distribution of how subjects were contacted and participation rate is systematically tracked. Interested participants complete a phone screen to confirm eligibility ask questions, discuss informed consent, and schedule his/her baseline visit.

Consented participants will be randomly assigned in a 1:1:1 ratio to one of the following programs: (1) community-based Qigong, (2) Internet-based Qigong, or (3) a self-care control group. Treatment assignments will be generated by a statistician not involved in analyses using a permuted block design. Randomization will be stratified by gender and age of caregiver (2 strata: 35–65 years; > 65 years). Assignments will be delivered to the study coordinator in sealed opaque envelopes and opened by them at the time of randomization. Neither participants nor research staff will be blinded to participants’ exposure. However, the statistician will score all the data without knowledge of group assignment and remain blinded until all statistical models are coded and finalized based on testing using dummy random codes for group assignment.

## Study intervention

### Eight Brocades

Qigong represents a pluralistic set of practices, not only including Tai Chi-like mind-body exercise regimens, but also stationary seated and standing meditative practices, and even laying of hands. We will evaluate one of the most widely practiced and scientifically evaluated medical Qigong regimens—namely, Eight Strands of the Brocades or Baduanjin (from here on referred to as “Eight Brocades”). Eight Brocades is easy to learn, has a well-documented history of use for health [47, 49, 51, 87–89], is considered a national exercise in China, and is widely available in the West. As a proxy for its popularity, Amazon.com lists 170 book or DVD products related to the Eight Brocades. Focus on this specific regimen will maximize the impact and generalizability of our proposed study findings.

Participants will engage in each movement of the Eight Brocades lead by the Qigong instructor. Each exercise involves slow flowing movements, deep rhythmic breathing, and a meditative state of mind. This class will include a 20-min warm up with stretching and breathing exercises followed by a 45-min session performing the Eight Brocades. The class will end with a 10-min cool down period that includes self-massage and meridian tapping. The name of each movement and the order in which they are presented can be found in Table 1.

**Table 1:**
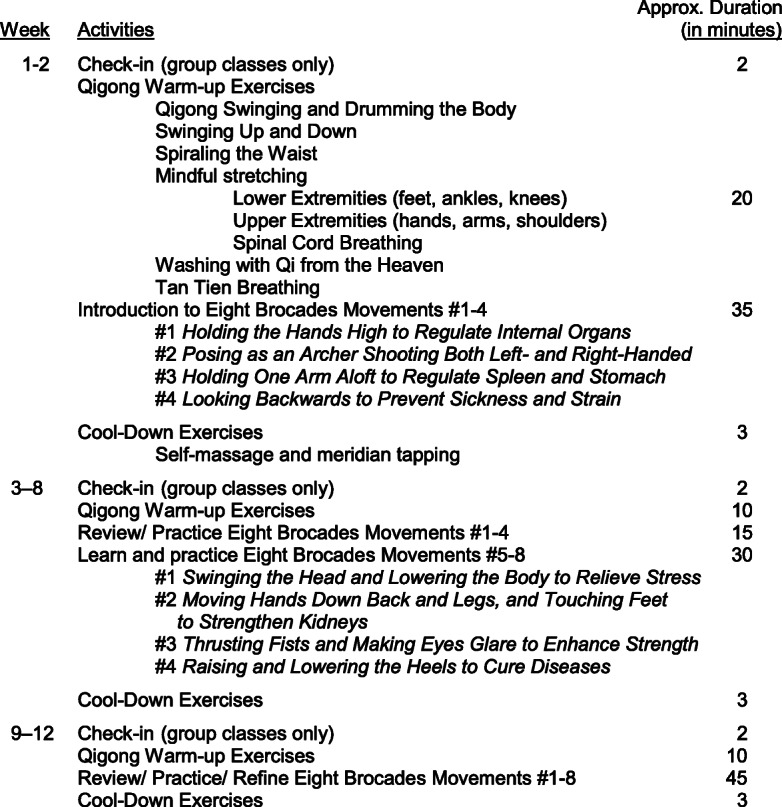
Outline for Eight Brocade Protocol

Both our community- and Internet-based Qigong programs will be based on the Eight Brocades system. Classes will be taught by instructors who are knowledgeable about working with cancer-related distress and have excellent fundamental skills. The study’s lead senior Qigong instructor received certification as a Mind Body Intervention Specialist from MD Anderson Cancer Center and is a Certified Medical Qigong Instructor with 11 years of experience teaching Qigong and Tai Chi. Additional guidance in the Qigong protocol development will be provided by the study co-principal investigator (PW) who has over 40 years of Qigong training experience.

### Study groups

#### Community-based group (CBG)

Participants randomized to the CBG will be asked to attend one 75-min long Qigong class per week for 12 weeks. All classes will take place at a private exercise studio in downtown Houston. Additionally, these participants will be asked to practice Qigong at home for 20 min a day, 3 days a week. We provide them with links to online-guided Qigong practices and printed materials to guide their home practice. In addition, we provide them with Qigong practice logs to track the daily practice time and track adverse events (AEs). Study staff call participants in the CBG once a week to monitor any AEs. The total time commitment if assigned to the CBG is approximately 30 h. Table 1 includes a model of CBG class sessions for each week of the intervention.

#### Internet-based group (IBG)

Participants randomized to the IBG will be provided with a computer tablet and a research coordinator-led tutorial on how to use the Qigong program. Participants in the IBG will be asked to follow two pre-recorded video online sessions for 40 min each, also supplemented by home practice for 20 min on 3 additional days. Each 40 min video consists of 10-min warm up with stretching and breathing exercises followed by a 25-min session performing the Eight Brocades. At the end of the class, there is a 5-min cool down period that includes self-massage and meridian tapping. All sessions can be completed at a time and location convenient to these participants. Study staff call participants in the IBG once a week to monitor AEs and compliance.

During weeks 1, 2, 4, and 6 of the program, participants in the IBG group also complete a one-to-one video call with the Qigong instructor. The purpose of these real-time/live virtual lessons is to give participants the opportunity to address any training-related questions or concerns and to receive personalized feedback and encouragement from the instructors. These sessions will also be used to identify, early on, any modifications required to address safety concerns. All virtual lessons will be digitally recorded. Review of these sessions provides qualitative insight into the experience of participants randomized into the Internet-delivered arm of the study and also informs discussions on optimization of this component of training. At the 12-week assessment, participants are asked to return the computer tablet. The total time commitment if assigned to the IBG is approximately 30 h.

#### Self-care group (SCG)

Participants randomized to the SCG will receive an educational book on caregiving that includes self-guided activities related to caregiving and caregiver health (*The Caregiver Helpbook: Powerful Tools for Caregiving*) [90]. The book’s evidence-based program is designed to provide caregivers the tools to increase their self-care and their confidence to handle difficult situations, emotions, and decisions. In addition, participants in this group receive a self-care manual with guided weekly action plans and activities that correspond to subject matter covered in the weekly readings. Participants in this group will turn in their self-care manual at the 12-week follow-up visit. Finally, study staff will call participants in the SCG once a week to monitor any AEs. If the book recommendations are followed weekly, the time commitment could be up to 30 h.

## Outcomes

### Overview of outcomes

As a pilot study, our primary outcomes center on the assessment of feasibility of participant recruitment and retention, class attendance and adherence out of class, and the evaluation of the feasibility of the web-based intervention and of collecting and managing data, and the suitability of questionnaires for this population. All outcomes will be assessed in-person at baseline, post-treatment (12 weeks), and 6 months post-treatment to evaluate the longer-term stability of outcomes. Collectively, feasibility and clinical outcomes, in combination with qualitative interview data (described below) conducted at 12 weeks, will be obtained to inform the design of a future fully powered trial (see Table 2).

**Table 2:** Outcome measures

Measure	Description
Quality of Life	• Promis-29 [67]. 29 items consisting of self-reported health measures in the domains of physical health, mental health and social health.
Depression	• Patient Health Questionnaire-9 (PHQ-9) [68]. 9 items measuring depression and used to grade severity of symptoms.
Fatigue	• Brief Fatigue Inventory (BFI) [69]. 9 items measuring the severity of fatigue and the impact of fatigue on daily functioning in the past 24 hours.
Caregiver Fear of Recurrence	• Fear of Recurrence-Caregiver version (FOR) [70, 71]. 22 items measuring the amount of worry and concern cancer caregivers have about the cancer recurring.
Anxiety	• Generalized Anxiety Disorder (GAD-7) [72, 73]. A 7-item tool used for screening, diagnosis and severity assessment of anxiety disorder.
Sleep Disturbances	• Pittsburgh Sleep Quality Index (PSQI) [74]. 19 items measuring patients’ sleep quality, sleep latency, sleep duration, habitual sleep efficiency, sleep disturbances, sleeping medication use, and daytime dysfunction over the past month.
Perceived Social Support	• Multidimensional Scale of Perceived Social Support (MSPSS) [75]. A 12-item scale designed to measure perceived social support from three sources; Family, Friends and a Significant Other.
Perceived Stress	• Perceived Stress Scale (PSS) [76]. A 10-item psychological instrument measuring the perception of stress.
Caregiver Burden	• Caregiver Burden Scale (CBS) [77]. 22 items measuring the impact of caregiving on three dimensions of burden: objective, subjective demand, and subjective stress.
Physical activity	• Godin Leisure-Time Exercise Questionnaire [78]. 4 items measuring the frequency of light-intensity, moderate-intensity, and vigorous-intensity leisure-time physical activity.
Exercise self-efficacy	• Self-Efficacy Scale [79]. 9 items measuring self-efficacy expectations related to the ability to continue exercising in the face of barriers to exercise.
Physical function^a^	
• Grip strength	• Strength of the dominant hand will be measured using a Jamar hydraulic hand dynamometer (Patterson Medical – Canada, Mississauga, ON, Canada) [80].
• Digit span	• Participant repeats a series of numbers the researcher says out loud; repetition is first forwards and then backwards. The trial is failed after two incorrect attempts in one test by the participant [81].
• Trail Making Test	• Participants complete tests A and B. For the Part A test the subject draws lines connecting circles containing the numbers 1–25 in ascending order; for Part B subject craws line connecting corresponding numbers and letters of the alphabet. Subject must not lift pen from paper or the trial is failed. Both trials are timed [82].
• Timed-Up-and-Go	• The Timed-Up-and-Go (TUG) test is a simple and widely used measure of mobility that measures the time it takes to stand up from a chair, walk 3 m, turn around an obstacle, walk back, and sit down [83].

^a^Physical function measures only assessed at baseline and 12 weeks

### Qigong practice logs

For the duration of the 12-week intervention, participants will be provided Qigong practice logs to record the number of times, specific day, and duration for which they practiced each week. Practice logs will be completed daily and submitted to the RA at the 12-week assessment. The study’s research assistant will remind subjects to complete the logs during weekly check-in calls.

### Additional patient-reported outcome measures

At baseline as well as at the 12-week and 6-month follow-up time points, participants will complete the following questionnaires.

- Promis-29: 29 items consisting of self-reported health measures in 7 key domains: physical function, anxiety, depression, fatigue, sleep disturbance, ability to participate in social roles and activities, pain interference, and pain intensity. All items except for a single question evaluating pain intensity will be rated on a 5-point Likert scale. PROMIS-29 has excellent psychometric properties and offers the ability to compare scores across conditions and to general population norms [67].
- Patient Health Questionnaire-9 (PHQ-9): 9 items measuring depression and asks how often respondents have been bothered by problems in the last 2 weeks. Items will be rated on a 4-point Likert-type scale, ranging from 0 (not at all) to 3 (nearly every day). Total score can range from 0 to 27, with higher scores indicative of more depression [68].
- Brief Fatigue Inventory (BFI): a 9-item, 11-point rating scale developed to assess subjective fatigue. The first three questions measure fatigue severity and the remaining six questions assess fatigue interference with daily activities. Higher scores on the BFI correspond to greater self-reported levels of fatigue. Reliability was excellent with an internal consistency coefficient of 0.96 [69].
- Fear of Recurrence-Caregiver version (FOR): 22 items measuring the amount of worry and concern cancer caregivers have about the cancer recurring. Higher scores indicate greater FOR [70, 71].
- Generalized Anxiety Disorder (GAD-7): a 7-item tool measuring worry and anxiety symptoms. Each item is scored on a four-point Likert scale (0–3) with total scores ranging from 0 to 21 with higher scores reflecting greater anxiety severity. The GAD-7 has shown good reliability and construct validity [72, 73].
- Pittsburgh Sleep Quality Index (PSQI): 19 items measuring patients’ sleep quality, sleep latency, sleep duration, habitual sleep efficiency, sleep disturbances, sleeping medication use, and daytime dysfunction over the past month. Each item is scored on a 4-point Likert scale (0 to 3), with a global sum of “5”or greater indicating a “poor” sleeper. The PSQI has internal consistency and a reliability coefficient of 0.83 for its seven components [74].
- Multidimensional Scale of Perceived Social Support (MSPSS): 12-item measure of perceived adequacy of social support from three sources: family, friends, & significant other; using a 5-point Likert scale (0 = strongly disagree, 5 = strongly agree) [75].
- Perceived Stress Scale (PSS): a 10-item psychological instrument measuring feelings and thoughts during the last month using a 5-point Likert scale. PSS scores will be obtained by reversing responses (e.g., 0 = 4, 1 = 3, 2 = 2, 3 = 1, and 4 = 0) to the four positively stated items (items 4, 5, 7, and 8) and then summing across all scale items [76].
- Caregiver Burden Scale (CBS): 22 items measuring the impact of caregiving on three dimensions of burden: objective, subjective demand, and subjective stress. Each item is scored on a 5-point Likert scale (0 to 4), with higher scores indicative of greater burden. The CBS has satisfactory validity and reliability with scores in the range of 0.89–1.0 [77].
- Godin Leisure-Time Exercise Questionnaire: 4 items measuring the frequency of light-intensity, moderate-intensity, and vigorous-intensity leisure-time physical activity during a 7-day period [78].
- Self-Efficacy for Exercise Scale (SEE): 9 items measuring self-efficacy expectations related to the ability to continue exercising in the face of barriers to exercise. The total score is calculated by finding the sum of all items. This scale has a range of total scores from 0 to 90. A higher score indicates higher self-efficacy for exercise. SES has an internal consistency of 0.92 [79].

We will be conducting 30 min, semi-structured, open-ended interviews at the 12-week follow-up with subjects randomized to the Qigong groups. Specifically, we will attain participants’ reasons for joining and remaining in the trial, expectations of the Qigong interventions, experience with and perceived effects of Qigong, and ease of practicing in the community class, at home, and with videos. Questions specific to the mode of intervention delivery may include those related to use of technology, the role of face-to-face instructor feedback, perceived motivators to adherence, and self-efficacy. Each interview will be audio-recorded, then transcribed verbatim. The qualitative data will be analyzed using grounded theory approach.

## Safety monitoring

### Risks of intervention

The risks of AEs and especially serious AEs associated with Qigong and related mind-body practices are generally believed to be very low [91]. Potential physical AEs include muscle soreness, shortness of breath, or dizziness from physical activity if the subject has not exercised in a long time. In order to mitigate these potential AEs, participants will be strongly encouraged to stretch before beginning any exercises, move at their own pace, rest when needed, and stop any particular exercise or movement if they feel it is causing them discomfort. Potential psychological AEs include feelings of discomfort, embarrassment, or stress that may arise as a result of filling out questionnaires or taking part in interviews. To mitigate the risks of these AEs, participants will be told they may discontinue the survey or interview at any time, or skip/neglect to answer any question that causes them distress.

### Adverse events monitoring and classification

A multi-pronged approach will be utilized to monitor safety and track AEs throughout the study with formal oversight by a Data and Safety Monitoring Committee. An AE is defined by our institution’s human subjects review board as an unwanted physical or psychological symptom or disease that occurs during the subject’s participation in the research, even if it is unrelated to the research. AEs will be classified as serious, mild, or non-serious; expected or unexpected; and definitely related to the intervention, probably related to the intervention, possibly related to the intervention, unlikely to be related to the intervention, or unrelated to the intervention. If any reported or observed AE is thought to be possibly related to the intervention, it will be reported to the IRB.

AEs will be logged by the research staff in several ways. For participants attending community classes, instructors will be trained to track and report any observed or participant-reported AE in hand-written logs. Participants assigned to both CBG and IBG will also be asked to complete an AE survey weekly, which will be sent to study staff. Participants in all three groups, including the SCC, will be called weekly to ask about any adverse events, and more generally, to maintain contact with study staff. Finally, all participants will be queried in-person at 12 weeks about AEs experienced over the course of the study. Reporting of AEs will follow institutional IRB guidelines.

## Analysis plan

### Evaluation of feasibility

For the primary aim, feasibility will be assessed with respect to participant recruitment, retention, intervention adherence (in class and out of class), intervention acceptability, and completion of outcome measures. Recruitment feasibility will be determined using total number of enrolling participants divided by total number of eligible participants (including participants refusing to enroll). Percentage of the eligible participants out of total screened participants will also be calculated. We will document reasons for refusal. These thresholds of feasibility will be as follows: (a) ≥ 50% screened individuals are study eligible and (b) ≥ 5% of eligible participants are willing to consent. With a goal of *n* = 54 enrolled over a 1-year period, we will need to screen 14 participants per month. Participant retention will be deemed feasible if loss to follow-up is < 20%. Intervention adherence will require: (a) ≥ 70% participation in community or Internet-guided classes and > 50% compliance with home practice guidelines. Qualitative interviews with cancer caregivers, including those who drop out and have low adherence, will be used to further inform overall study feasibility, and facilitators and barriers to participation.

According to the above criteria, the frequency and rate of retention as well as adherence in each treatment group will be obtained and compared using cross-tabulations with Pearson’s chi-square or Fisher’s exact tests. Furthermore, attendance will be recorded as the proportion of classes attended for community-based classes, and online video tutorial use, logs of home practice, numbers of logins to the webpage, clicks on Qigong video links, downloads, and views of videos for the Internet-based classes. Similar criteria will be set for Internet-based compliance as well as home practice compliance in both groups.

For the evaluation of AEs, we will compare the frequency of all AEs and severe treatment-emergent adverse events between the treatment groups using negative binomial regression. For some AEs that contain excess zeros (the AEs are rare), then the zero-inflated negative binomial regression will be performed instead. Additionally, time to report such rare adverse event for the first time will be created and compared via log-rank test and expressed with survival curves.

### Clinical study endpoints

We will summarize the baseline characteristics of those randomized to the intervention groups versus those randomized to the self-care group using means and standard deviations or medians and interquartile ranges for continuous variables and frequencies and percentages for dichotomous or categorical variables.

As a pilot study, we did not plan to test for efficacy but rather to obtain information on the feasibility of our design and effective size estimates. We will estimate within group changes and variability, preliminary sense of utility and effective size estimates, and sensitivity of the outcomes to inform which to focus on in future studies. The study clinical outcomes to be assessed were chosen to reflect a broad range of psychological and physical factors affected by cancer caregiver burden and distress. At the initial study visit, assessment related to QOL, psychological well-being, caregiver burden, and physical function will be measured. An intention-to-treat method will be used to estimate the benefits of the changes in the treatment. We will use linear mixed models with fixed effects of visit time, treatment × post-baseline visit interaction, and unstructured covariance among repeated measures to analyze clinical outcomes. The Wald statistic from the treatment × visit interaction testing 12-week treatment-dependent response will be used to estimate the effect of Qigong and as a criterion for selecting outcomes that are potentially sensitive to Qigong. Furthermore, sensitivity analyses will be conducted using perturbed multiple imputation if the drop-out rates strongly depend on the treatment effects.

### Sample size justification

We expect to consent, enroll, and randomize a total of 54 caregivers. The sample size was based on practical/budgetary constraints as well as a preliminary power analysis. A power analysis was conducted to determine the minimum sample size that is required to find significance with a power set of .80, an alpha level at .05, correlation among the repeated measures of 0.3, and a moderate effect size of .25 using G*Power version 3.1. Based on the primary analysis, to ensure sufficient power on the repeated measure ANOVA (3 time points × 3 groups), a total of 48 participants will be required. In consideration of 15% of attrition rate, a total of 55 participants will be recruited.

### Qualitative data

Qualitative interviews with cancer caregivers, including those who drop out and have low adherence if possible, will be used to further inform overall study feasibility, and facilitators and barriers to participation. These interviews will be analyzed using a grounded theory methodology, where a researcher forms theories after the data has been transcribed and analyzed. Emergent themes will be coded and identified by two independent researchers using a process of constant comparison. Information from the interview transcripts will be extracted and then emergent themes discussed. Using these themes, the researchers will create categories and subcategories, which will be used to analyze the next round of interview transcripts. At least 8–10 participants in each group will be interviewed and the data collection will be continuously gathered until the thematic saturation is reached. Transcription, coding, and analysis will be conducted in NVivo v11.

## Data management

Study data from paper forms will be entered into REDCap (Research Electronic Data Capture) tools. REDCap provides a secure, web-based interface for validated data entry with auditing features for tracking data manipulation, and export of data to common statistical packages and data importation from external data sources. All data entered will be cross-checked and the database will contain range and logic checks in order to minimize errors.

## Discussion

This 3-arm RCT will be the first to evaluate the psychosocial and physical health benefits of the Eight Brocades Qigong intervention for caregivers of cancer patients. As the numbers of survivors and caregivers increase, Internet-based interventions to support this population to decrease burden and enhance QOL is warranted. Our pilot study, and our eventual large-scale comparative effectiveness trial, explores the effectiveness of Qigong training delivered in both community-based group classes and through self-guided Internet-based modules supplemented with one-on-one virtual learning support. A finding that both methods are effective would lead to multiple options for caregivers. Those with flexible schedules and seeking group support and the opportunity for a change of environments might choose community classes. Those with unpredictable and inflexible schedules and an inability to leave the home could develop a self-guided training plan, with one-on-one virtual support scheduled at a convenient time. More generally, the evidence base for DVD- and Internet-based learning of mind-body exercise is essentially non-existent. Studies are needed to evaluate whether the promising evidence of Qigong’s effectiveness based on group and instructor-led clinical trials is also observed when instructions are delivered virtually.

## Supplementary Information

**Additional file 1.**

## Data Availability

The corresponding author will make the final de-identified data from this study available upon request.
