# Supplementary material for: Qigong mind-body program for caregivers of cancer patients: design of a pilot three-arm randomized clinical trial
Source: Pilot Feasibility Stud. 2021 Mar 19;7:73. doi: 10.1186/s40814-021-00793-4 (PMC7976717; doi:10.1186/s40814-021-00793-4)
Supplement: Supplementary file 1 [file 40814_2021_793_MOESM1_ESM.docx]

Appendix

The Caregiver Helpbook: Powerful Tools for Caregiving

Self-Care Manual

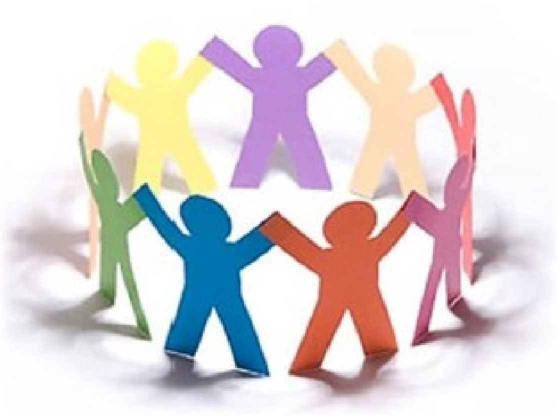

TABLE OF CONTENTS

The Caregiver Helpbook: Powerful Tools for Caregiving
Self-Care Manual

SAMPLE ACTION PLAN

WEEK 1
CHAPTER 1, TAKING CARE OF YOU (READ 1-11)

WEEK 2
CHAPTER 2, REDUCING PERSONAL STRESS WITH POSITIVE AFFIRMATIONS (READ 15-27)

WEEK 3
CHAPTER 2, REDUCING PERSONAL STRESS (READ 28-31 AND 34-37)

WEEK 4
CHAPTER 3, COMMUNICATING EFFECTIVELY WITH OTHERS, BETTER LISTENING AND EXPRESSING YOURSELF (READ 39-50)

WEEK 5
CHAPTER 3, COMMUNICATING EFFECTIVELY WITH OTHERS, COMMUNICATION STYLES (READ 50-59)

WEEK 6
CHAPTER 4, COMMUNICATING EFFECTIVELY IN CHALLENGING SITUATIONS (READ 61-87)

WEEK 7
CHAPTER 5, LEARNING FROM OUR EMOTIONS- FEELINGS AND CAREGIVING (READ 89-115)

WEEK 8
CHAPTER 6, MASTERING CAREGIVING TRANSITIONS (READ 115-132)

WEEK 9
CHAPTER 9, USING COMMUNITY SERVICES (READ 179-195)

WEEK 10
CHAPTER 12, HELPING A FAMILY MEMBER HANDLE FINANCES (READ 249-259)

WEEK 11
CHAPTER 13, RECOGNIZING AND RESPONDING TO DEPRESSION (READ 261-283)

WEEK 12
SELF-REFLECTION ACTIVITY

CAREGIVER ACTION PLAN – ***Sample***

Create an action plan that you are confident you can accomplish *within the next week*.

1. **What** you are going to do?
2. **Amount of time** you are going to do it (estimated length of time spent on the chosen activity).
3. **When** you are going to it, (at what time of day will you do it)?
4. **How often** you will do it (once per day, twice per week, or some other frequency).

Example: This week I will go on a relaxing walk (what) for at least a half hour (how much) in the mid-afternoon when my husband sleeps (when), three times- Monday, Wednesday and Friday (how often).

This week I will go for a walk. (what)

This week I will walk for at least half an hour. (how much)

I will walk during the mid-afternoon, when my husband sleeps. (when)

I plan to walk three days this week- Monday, Wednesday and Friday. (how often)

How confident are you that you will complete your entire action plan during the week?

Not at all Confident — Very Confident

(Circle) 0 1 2 3 4 5 6 (7) 8 9 10

Check off each day you accomplish your plan		Comments:
Monday	__X_	My walk was very refreshing.
Tuesday	_____	
Wednesday	_____	My niece came over and we had a great visit.
Thursday	__X_	Walked for 45 minutes; it was stimulating.
Friday	__X_	Walked for 15 minutes, but my husband called and needed me at home.
Saturday	__X_	Went on an early morning walk in neighborhood; a great start to my day.
Sunday	_____	

Weekly Question:

Was the content of this week's chapter helpful to you for increasing your self-care? Please circle one option below.				
Strongly Disagree	Disagree	Neutral	Agree	Strongly Agree

Adapted from The Caregiver Helpbook: Powerful Tools for Caregiving, by Vicki L. Schmall, PH.D., Marilyn Cleland, R.N. and Marilynn Sturdevant, R.N., M.S.W., L.C.S.W.

CAREGIVER ACTION PLAN – WEEK 1

Focus: Chapter 1, Taking Care of You (read pg. 1-11 and respond below)

Create an action plan that you are confident you can accomplish *within the next week*; also see sample action plan at the beginning of the manual.

1. **What** you are going to do.
2. **How** much you are going to do.
3. **When** you are going to do it (i.e. what time of day)
4. **How often** you are going to do it.

This week I will____(what)

____(how much)

____(when)

____(how often)

How confident are you that you will complete your entire action plan during the week?

Not at all Confident Very Confident

(Circle) 0 1 2 3 4 5 6 7 8 9 10

Check off each day you accomplish your activity:		Comments:
Monday	____	____________________
Tuesday	____	____________________
Wednesday	____	____________________
Thursday	____	____________________
Friday	____	____________________
Saturday	____	____________________
Sunday	____	____________________

Weekly Question:

Was the content of this week's chapter helpful to you for increasing your self-care? Please circle one option below.				
Strongly Disagree	Disagree	Neutral	Agree	Strongly Agree

CAREGIVER ACTION PLAN – WEEK 2

Focus: Chapter 2, Reducing Personal Stress with Positive Affirmations (read pg. 15-27 and respond below)

Create an action plan to challenge negative self-talk by identifying an example of negative self-talk and the situation when it is most likely to occur. Be clear and as detailed as possible; write it down. Record days/times when you chose a positive affirmation, instead of engaging in negative self-talk.

1. **Example** of your negative self-talk:

2. I say the statements above to myself **when**:

3. I will **replace** this negative thought with this positive statement:

4. **Repeat the chosen affirmation** whenever the above situation occurs and record the instances of success below.

How confident are you that you will complete your action plan during the week?

Not at all Confident Very Confident

(Circle) 0 1 2 3 4 5 6 7 8 9 10

Check off day you used affirmations		Comments
Monday	_____	______________________________
Tuesday	_____	______________________________
Wednesday	_____	______________________________
Thursday	_____	______________________________
Friday	_____	______________________________
Saturday	_____	______________________________
Sunday	_____	______________________________

Weekly Question:

Was the content of this week's chapter helpful to you for increasing positive affirmations? Please circle one option below.				
Strongly Disagree	Disagree	Neutral	Agree	Strongly Agree

CAREGIVER ACTION PLAN – WEEK 3

Focus: Chapter 2, Reducing Personal Stress- (read pg. 28-31 and 34-37; respond below)

Identify **one** of your personal stressors and develop an action plan for addressing this stressor ***this week***. Below, write down the stressor you have identified. Create an action plan to help you address this source of stress.

1. **What** is the stressor you identified?
2. **What can I do** about the stressor (ignore, change perception, use positive self-talk, etc.)
3. **When** will you take to address this stressor? Record instances below.
4. **How often** do you anticipate this happening?

This week I will____(what can I do)

____(what/the stressor)

____(whenever…)

____(how often)

How confident are you that you will carry out your action plan during the week?

Not at all Confident — Very Confident

(Circle) 0 1 2 3 4 5 6 7 8 9 10

Check off day you addressed your stressor		Comments
Monday	____	____________________
Tuesday	____	____________________
Wednesday	____	____________________
Thursday	____	____________________
Friday	____	____________________
Saturday	____	____________________
Sunday	____	____________________

Weekly Question:

Was the content of this week's chapter helpful to you for addressing a source of stress? Please circle one option below.				
Strongly Disagree	Disagree	Neutral	Agree	Strongly Agree

CAREGIVER ACTION PLAN – WEEK 4

Focus: Chapter 3, Communicating Effectively with Others, Better Listening and Expressing Yourself (read pg. 39-50 and respond below)

Using one of the strategies mentioned on pg. 47 in the section titled, "How to Best Express Yourself" choose **one** of the four tools to help you express yourself; create this week's action plan around your choice.

1. **What** tool will you use (using "I" messages, respecting the rights or feelings of other people by what you say or do, speaking directly to the person(s) involved, be a good listener).
2. **How** are you going to do it (what mental and/or verbal strategy will you use).
3. **When** you are going to do it (what situations)
4. **How often** you are going to do it (i.e. can you anticipate situations you will need to use this strategy)?

This week I will______________________________(what step you chose)

______________________________(how will you do it)

______________________________(when)

______________________________(how often)

How confident are you that you will carry out your action plan during the week?

Not at all Confident Very Confident

(Circle) 0 1 2 3 4 5 6 7 8 9 10

Check off each day you felt communication was clearer		Comments:
Monday	____	______________________________
Tuesday	____	______________________________
Wednesday	____	______________________________
Thursday	____	______________________________
Friday	____	______________________________
Saturday	____	______________________________
Sunday	____	______________________________

Weekly Question:

Was the content of this week's chapter helpful in assisting you with more clearly expressing yourself? Please circle one option below.				
Strongly Disagree	Disagree	Neutral	Agree	Strongly Agree

CAREGIVER ACTION PLAN – WEEK 5

Focus: Chapter 3, Communicating Effectively with Others, Communication Styles (read pg. 50-59 and respond below)

Using the Aikido style of communication (pg. 55-58) choose one of the four steps to build harmonious communication and create this week's action plan around your choice.

1. **What** step will you use (align, agree, redirect energies or resolve problems).
2. **How** are you going to do it (what mental and verbal strategies will you use).
3. **When** you are going to do it (what situations)
4. **How often** you are going to do it (i.e. can you anticipate situations you will need to use this communication style)?

This week I will____________________________________(what step you chose)

____(how will you do it)

____(when)

____(how often)

How confident are you that you will carry out you action plan during the week?

Not at all Confident Very Confident

(Circle) 0 1 2 3 4 5 6 7 8 9 10

Check off each day you felt communication improved		Comments:
Monday	____	____________________
Tuesday	____	____________________
Wednesday	____	____________________
Thursday	____	____________________
Friday	____	____________________
Saturday	____	____________________
Sunday	____	____________________

Weekly Question:

Was the content of this week's chapter helpful to you for building harmonious communication? Please circle one option below.				
Strongly Disagree	Disagree	Neutral	Agree	Strongly Agree

CAREGIVER ACTION PLAN – WEEK 6

Focus: Chapter 4, Communicating Effectively in Challenging Situations (read pg. 61-87 and respond below)

Continue to refine your communication skills using a strategy of your choice from this chapter (whatever may be most applicable to your situation as a caregiver).

1. **What** communication strategy you will use:
2. **With whom** are you going to use this strategy:
3. **When** you are going to do it.

This week I will____(what strategy)

____(with whom)

____(when)

How confident are you that you will carry out your action plan during the week?

Not at all Confident — Very Confident

(Circle) 0 1 2 3 4 5 6 7 8 9 10

Check off each day you used your strategy		Comments:
Monday	____	____________________
Tuesday	____	____________________
Wednesday	____	____________________
Thursday	____	____________________
Friday	____	____________________
Saturday	____	____________________
Sunday	____	____________________

Weekly Question:

Was the content of this week's chapter helpful to you for improving your communication skills? Please circle one option below.				
Strongly Disagree	Disagree	Neutral	Agree	Strongly Agree

CAREGIVER ACTION PLAN – WEEK 7

Focus: Chapter 5, Learning from Our Emotions- Feelings and Caregiving (read pg. 89-115 and respond below)

Feeling intense emotions as a caregiver is a normal and natural response to your situation. Choose one of the tools provided from the list on pages 111-112 in the book to develop this week's action plan (the tool you choose can be a tool you used in a previous action plan and want to continue to use/work on).

1. **What** tool you will use:
2. **With whom** are you going to use this strategy:
3. **When** you are going to do it.

This week I will____(what tool)

____(with whom)

____(when)

How confident are you that you will carry out your action plan during the week?

Not at all Confident   Very Confident

(Circle) 0 1 2 3 4 5 6 7 8 9 10

Check off each day you used your tool		Comments:
Monday	_____	____________________
Tuesday	_____	____________________
Wednesday	_____	____________________
Thursday	_____	____________________
Friday	_____	____________________
Saturday	_____	____________________
Sunday	_____	____________________

Weekly Question:

Was the content of this week's chapter helpful to you for learning to care for yourself/deal with emotions?<br>Please circle one option below.				
Strongly Disagree	Disagree	Neutral	Agree	Strongly Agree

CAREGIVER ACTION PLAN – WEEK 8

Focus: Chapter 6, Mastering Caregiving Transitions (read pg. 115-132 and respond below)

Understanding the processes of transition and change can help you develop your ability to master a transition. One tool this chapter focuses on to master periods of change is the power of a positive attitude. Choose a strategy from those mentioned on pg. 125-129 to create your action plan around this week.

1. **What** tool you will use:
2. **With whom** are you going to use this strategy:
3. **When** you are going to do it.

This week I will____(what tool)

____(with whom)

____(when)

How confident are you that you will carry out your action plan during the week?

Not at all Confident Very Confident

(Circle) 0 1 2 3 4 5 6 7 8 9 10

Check off each day you used your tool		Comments:
Monday	____	________________________
Tuesday	____	________________________
Wednesday	____	________________________
Thursday	____	________________________
Friday	____	________________________
Saturday	____	________________________
Sunday	____	________________________

Weekly Question:

Was the content of this week's chapter helpful to you in cultivating a positive mindset?<br>Please circle one option below.				
Strongly Disagree	Disagree	Neutral	Agree	Strongly Agree

CAREGIVER ACTION PLAN – WEEK 9

Focus: Chapter 9, Using Community Services (read pg. 179-195 and respond below)

Many caregivers feel they must do everything for the person in their care. However, there may be community resources in your area who can offer support. Based on the information in the chapter, please research and identify three community resources that might be beneficial to you based on your current situation as a caregiver:

Name of Organization	Contact Information	Purpose of the Organization	How this organization can help me

How difficult was it for you to locate relevant community resources for your situation/needs?

Not at all Difficult   Very Difficult

(Circle) 0 1 2 3 4 5 6 7 8 9 10

What days/times do you need assistance   Comments:

Monday ____ ______________________________

Tuesday ____ ______________________________

Wednesday ____ ______________________________

Thursday ____ ______________________________

Friday ____ ______________________________

Saturday ____ ______________________________

Sunday ____ ______________________________

Weekly Question:

Was the content of this week's chapter helpful to you in locating useful community resources? Please circle one option below.				
Strongly Disagree	Disagree	Neutral	Agree	Strongly Agree

CAREGIVER ACTION PLAN – WEEK 10

Focus: Chapter 12, Helping a Family Member Handle Finances (read pg. 249-259 and respond below)

Older people sometimes need assistance with managing their finances. This chapter discusses the guidelines for speaking about finances with the person in your care. Based on the information in the chapter, please identify three strategies you can use to discuss finances with the person in your care. If you have already discussed finances and found strategies that worked for you, please explain them below (see example).

Strategy when Discussing Finances	How you used the Strategy	Outcome
Example: Anticipating my Father's response, "All you care about is my money?"	*Example: Role-played the conversation with my sister and practiced addressing my concerns and not getting defensive*	*Example: Spoke with my father and kept the conversation on topic to address my concerns/his needs without arguing. He accepted my help.*

How difficult is it/was it for you to speak with the person in your care about finances?

Not at all Difficult Very Difficult

(Circle) 0 1 2 3 4 5 6 7 8 9 10

Weekly Question:

Was the content of this week's chapter helpful to you in speaking to the person in your care about finances? Please circle one option below.				
Strongly Disagree	Disagree	Neutral	Agree	Strongly Agree

CAREGIVER ACTION PLAN – WEEK 11

Focus: Chapter 13, Recognizing and Responding to Depression (read pg. 261-283 and respond below)

As a caregiver, you may be caring for a person who is depressed. Remember that you do not need to solve the problem alone. Based on the information in the chapter, please research and identify three community resources available to you who can provide professional help for people with depression:

Name of Organization	Contact Information	What services for depression does this organization provide:

How difficult was it for you to locate community resources that provide professional help for those with depression in your community?

Not at all Difficult Very Difficult

(Circle) 0 1 2 3 4 5 6 7 8 9 10

Weekly Question:

Was the content of this week's chapter helpful to you in locating community resources that provide professional help for those with depression? Please circle one option below.				
Strongly Disagree	Disagree	Neutral	Agree	Strongly Agree

CAREGIVER ACTION PLAN – WEEK 12

Focus: Self Reflection Activity

Create an action plan around **one** concept that you found *most useful* from the self-care book and would like to adopt into your daily routine moving forward.

1. **What** you are going to do.
2. **How** much you are going to do.
3. **When** you are going to do it (i.e. what time of day)
4. **How often** you are going to do it.

This week I will____(what)

____(how much)

____(when)

____(how often)

How confident are you that you will continue to adopt this behavior/mind-set moving forward?

Not at all Confident Very Confident

(Circle) 0 1 2 3 4 5 6 7 8 9 10

Check off each day you accomplish your activity/ mental/emotional goal: Comments:

Monday ____ ________________________________

Tuesday ____ ________________________________

Wednesday ____ ________________________________

Thursday ____ ________________________________

Friday ____ ________________________________

Saturday ____ ________________________________

Sunday ____ ________________________________

Weekly Question:

Was the content of this workbook and manual helpful to you for increasing your self-care and confidence to handle difficult situations/emotions/decisions as a caregiver? Please circle one option below.				
Strongly Disagree	Disagree	Neutral	Agree	Strongly Agree
